# Supplementary material for: Interleukin-6 Induces Stem Cell Propagation through Liaison with the Sortilin–Progranulin Axis in Breast Cancer
Source: Cancers (Basel). 2023 Dec 8;15(24):5757. doi: 10.3390/cancers15245757 (PMC10741783; doi:10.3390/cancers15245757)
Supplement: Supplementary file 1 [file cancers-15-05757-s001.zip › cancers-2580113-Supl File S2.pdf]

Article

Interleukin-6 induces stem cell propagation through liaison with the sortilin-progranulin axis in breast cancer

Supplemental figures

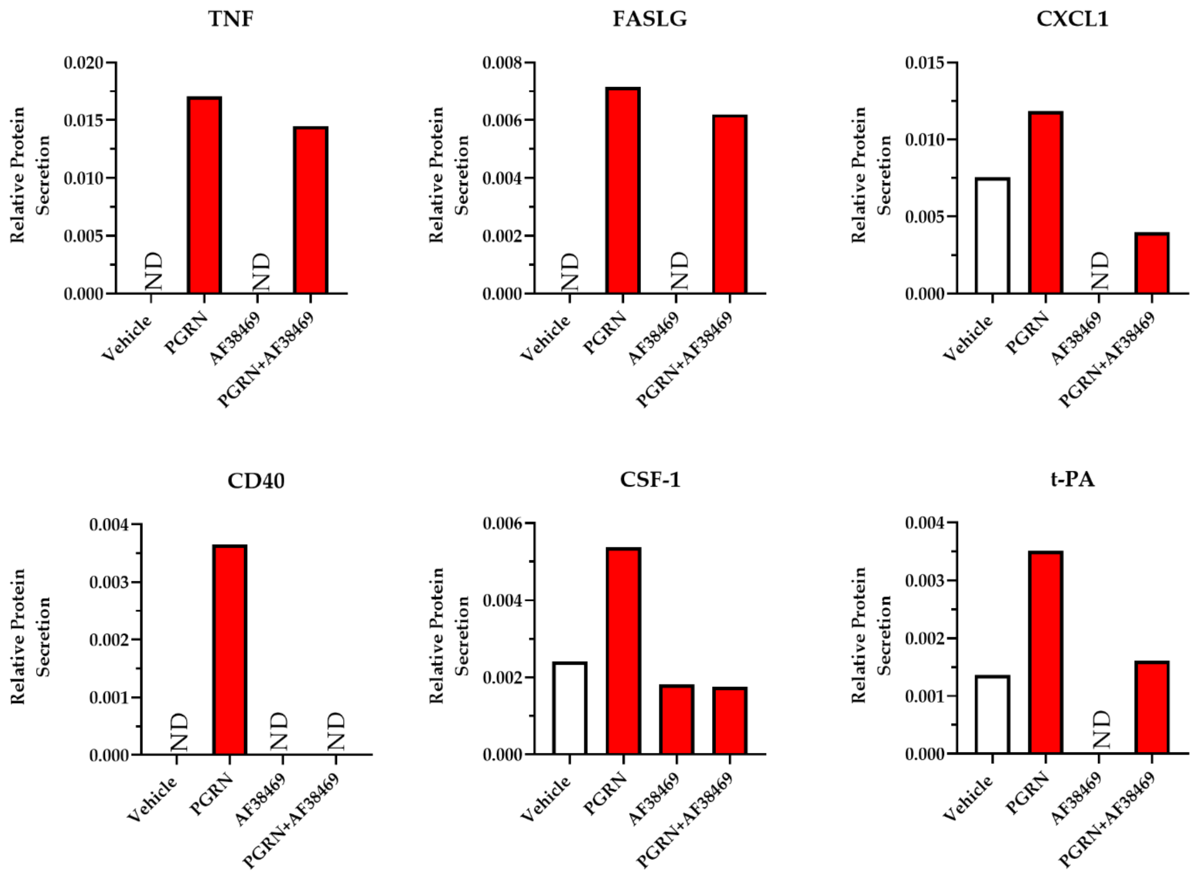

Figure S1. Changes in protein secretion after treatment with progranulin and AF38469 in MCF7 cells. MCF7 cells were untreated (vehicle), treated with 1 µg/ml progranulin or 2 µg/ml AF38469, or a combination, for 48 h. Bar charts showing increased secretion of TNF, FASLG, CXCL1, CD40, CSF-1 and t-PA in MCF7 cells treated with progranulin. Protein secretion was reduced in CXCL1, CD40, CSF-1 and t-Pa after treatment with AF38469. Data includes one biological replicate, ND: not detected, PGRN: progranulin, CXCL1: Chemokine (C-X-C motif) ligand 1, CD40: cluster of differentiation 40, TNF: tumor necrosis factor, FASL: tumor necrosis factor ligand superfamily member 6, CSF-1: colony stimulating factor 1, tPa: tissue plasminogen activator.

(a)

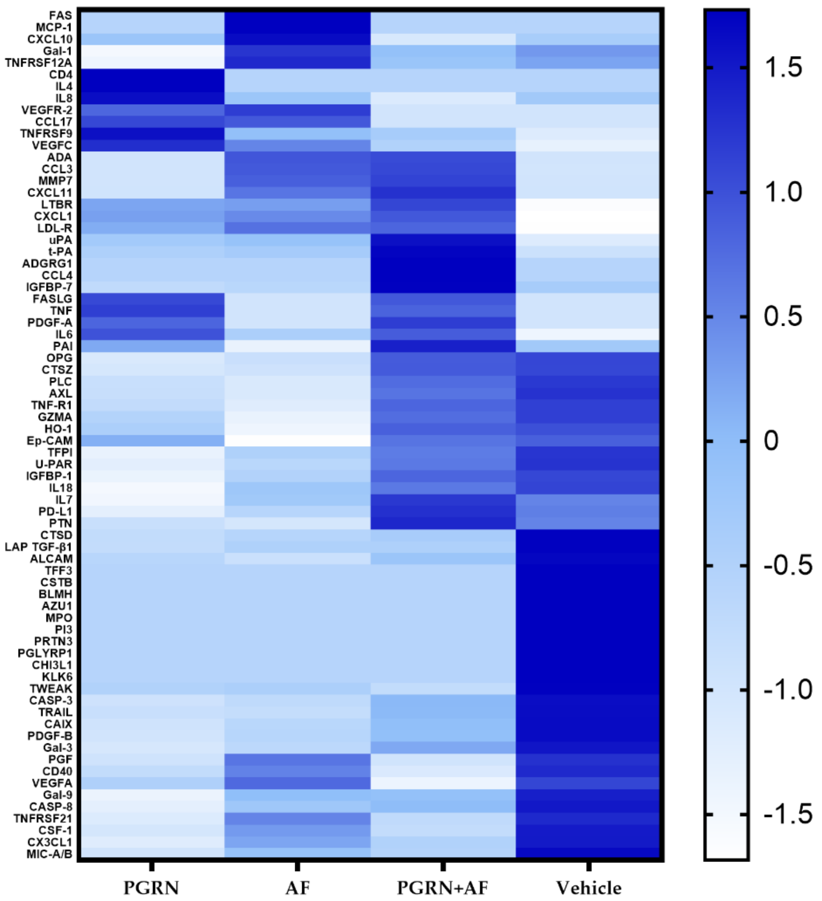

(b)

**Highly secreted proteins with progranulin treatment**
CD4
IL-4
IL-8
CCL17
TNFRSF9
VEGFC
CXCL1
FASL
TNF
IL-6

Figure S2. Progranulin treatment increased IL-6 and IL-8 secretion in breast cancer cells. (a) Heat map showing secreted proteins and cytokines in MDA-MB-231 cells. The cells were untreated (Vehicle), treated with 1 μg/ml progranulin or 2 μg/ml AF38469, or a combination, for 48 h. The data is auto-scaled and each protein is normalized to the total secretion of each sample. (b) Table of highly secreted proteins with progranulin treatment. Data represents one biological replicate. PGRN: progranulin, IL: interleukin.

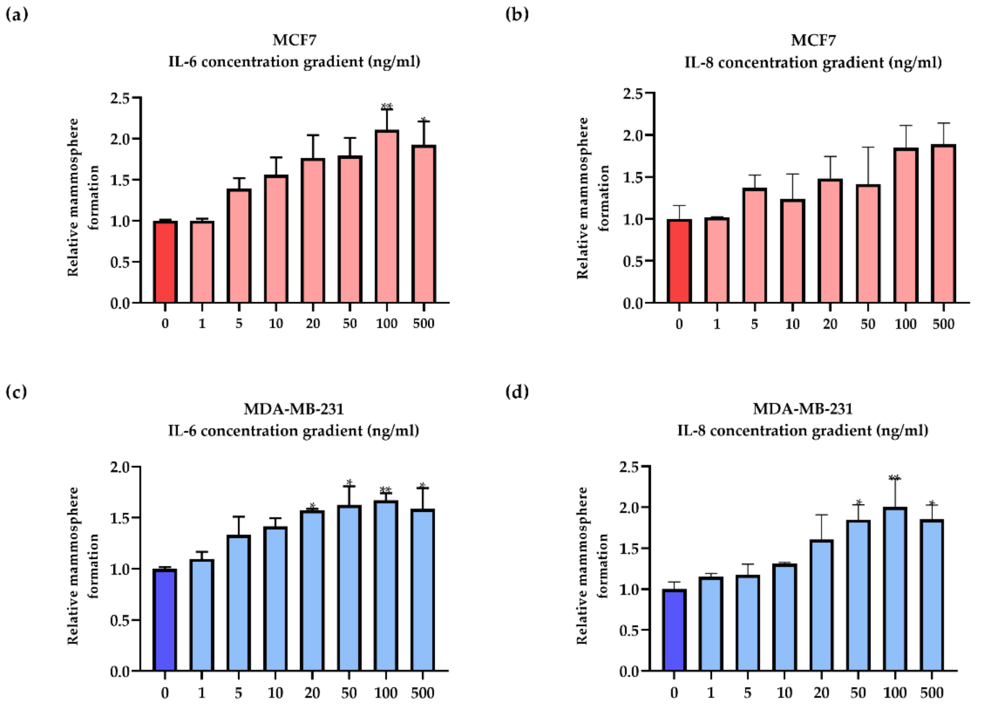

Figure S3. IL-6 and IL-8 induced mammosphere formation in MCF7 and MDA-MB-231 cells. Mammosphere-forming capacity in (a, b) MCF7 and (c, d) MDA-MB-231 cells treated with IL-6 or IL-8 for 48 h. The bar charts represent the relative number of spheres formed in control samples against various concentrations of IL-6 or IL-8, five days after cell seeding in non-adherent polyHEMA-coated 6-well plates. Results are presented as relative mammosphere formation ± SEM normalized to the control (0 ng/ml). Statistical significance was calculated using one-way ANOVA adjusted for multiple comparison (n=3), where $^*P < 0.05$ and $^{**}P < 0.01$ IL: interleukin.

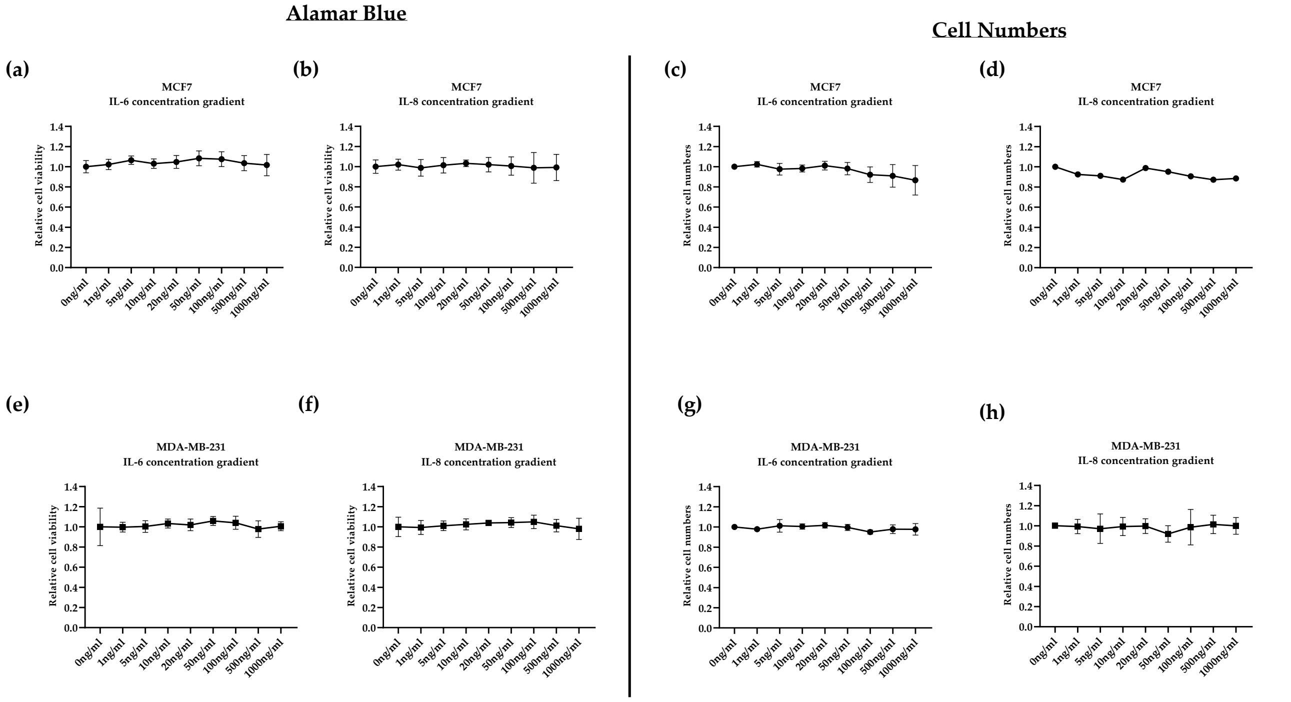

Figure S4. Alamar blue viability assay. Dose response curves of IL-6 and IL-8 treatment for 48 h were generated using the Alamar Blue assay or absolute cell count for (a, b, c, d) MCF7 and (e, f, g ,h) MDA-MB-231 cells. Values are represented relative to proliferation status before treatment and to the control (0 ng/ml). No significant difference in viability could be detected. Data is represented as mean ± SEM (n=3-4).

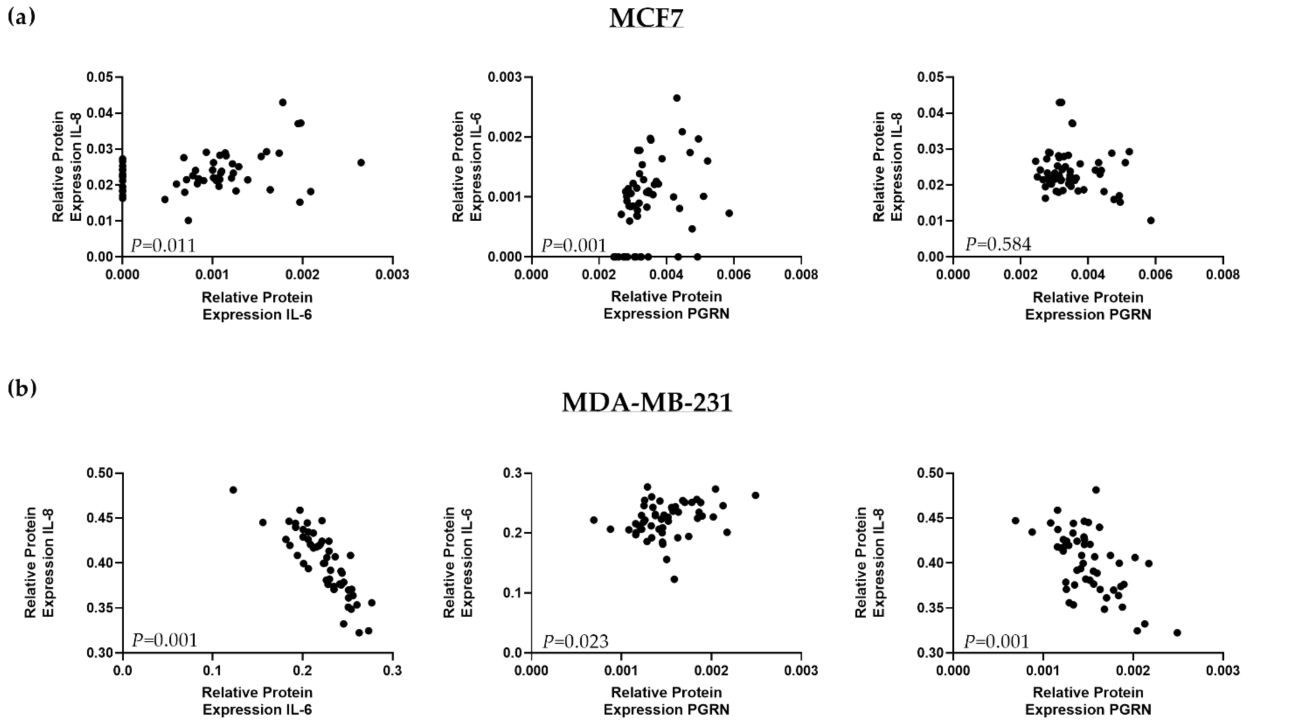

Figure S5. Scatterplot of the correlation between secreted IL-6, IL-8 and progranulin in the PDS model system. *P*-values calculated using Spearman correlation. $P < 0.05$ is considered statistically significant. For MCF7cells grown on patient-derived scaffolds, n=57 and for MDA-MB-231 cells grown on patient-derived scaffolds, n=53. PGRN: progranulin, IL-6: interleukin-6, IL-8: interleukin-8.

Table S1. Amino acid sequence of synthesized secreted sortilin (sSORT).

Amino acid sequence of secreted sortilin, after removal of signal peptide and pro-peptide (N-terminal is not confirmed experimentally).	SAPGEDEECGRVRDFVAKLANNTHQHVFDDLRGSVSLSWVGDSTGVILVLTTFHVPLVIMT FGQSKLYRSEDYGKNFKDITDLINNTFIRTEFGMAIGPENSGKVVLTAEVSGGSRGGRIFRSSD FAKNFVQTDLPFHPLTQMMYSPQNSDYLLALSTENGLWVSKNFGGKWEEIHKAVCLAKWG SDNTIFFTTYANGSCKADLGALELWRTSDLGKSFKTIGVKIYSFGLGGRFLFASVMADKDTTRR IHVSTDQGDTWSMAQLPSVGQEQFYSILAANDDMVFMHVDEPGDTGFGTIFTSDDRGIVYS KSLDRHLYTTTGGETDFTNVTSLRGVYITSVLSEDNSIQTMITFDQGGRWTHLRKPENSECDAT AKNKNECSLHIHASYSISQKLNVPMAPLSEPNAVGIVIAHGSVGDAISVMVPDVYISDDGGYS WTKMLEGPHYYTILDSGGIIVAIEHSSRPINVIKFSTDEGQCWQTYTFTRDPIYFTGLASEPGAR SMNISIWGFTESFLTSQWVSYTIDFKDILERNCEEKDYTIWLAHSTDPEDYEDGCILGYKEQFLR LRKSSVCQNGRDYVVTKQPSICLCSLEDFLCDFGYYRPENDSKCVEQPELKGHDLEFCLYGREE HLTTNGYRKIPGDKCQGGVN PVREVKDLKKKCTSNFLSPEKQNSKSNSTGHHHHHH
