# Supplementary material for: Interleukin-6 Induces Stem Cell Propagation through Liaison with the Sortilin–Progranulin Axis in Breast Cancer
Source: Cancers (Basel). 2023 Dec 8;15(24):5757. doi: 10.3390/cancers15245757 (PMC10741783; doi:10.3390/cancers15245757)
Supplement: Supplementary file 1 [file cancers-15-05757-s001.zip › cancers-2580113-suppl. file S1.pdf]

Article

Interleukin-6 induces stem cell propagation through liaison with the sortilin-progranulin axis in breast cancer

Figure 2, supporting file: Whole gel WB images.

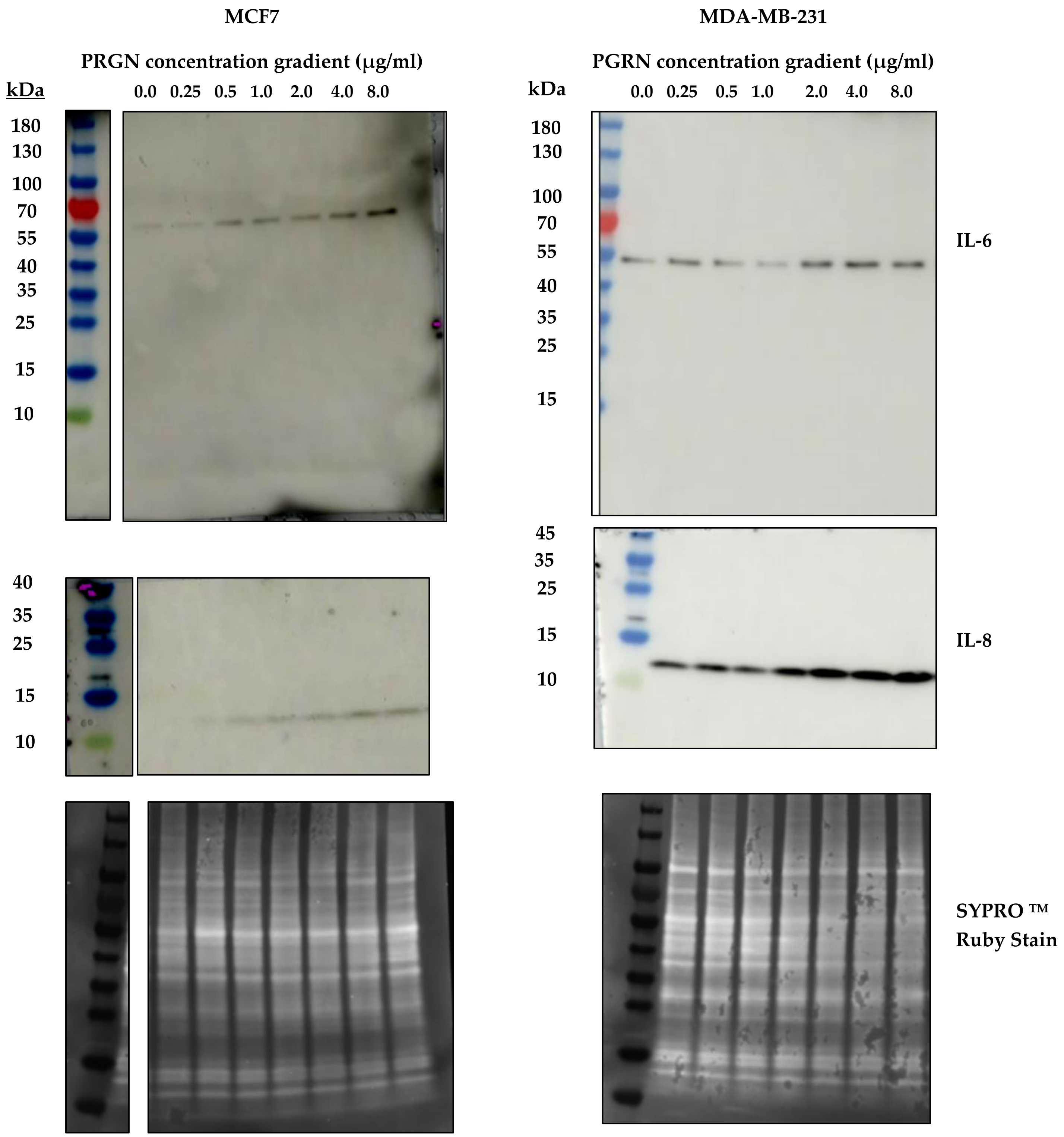

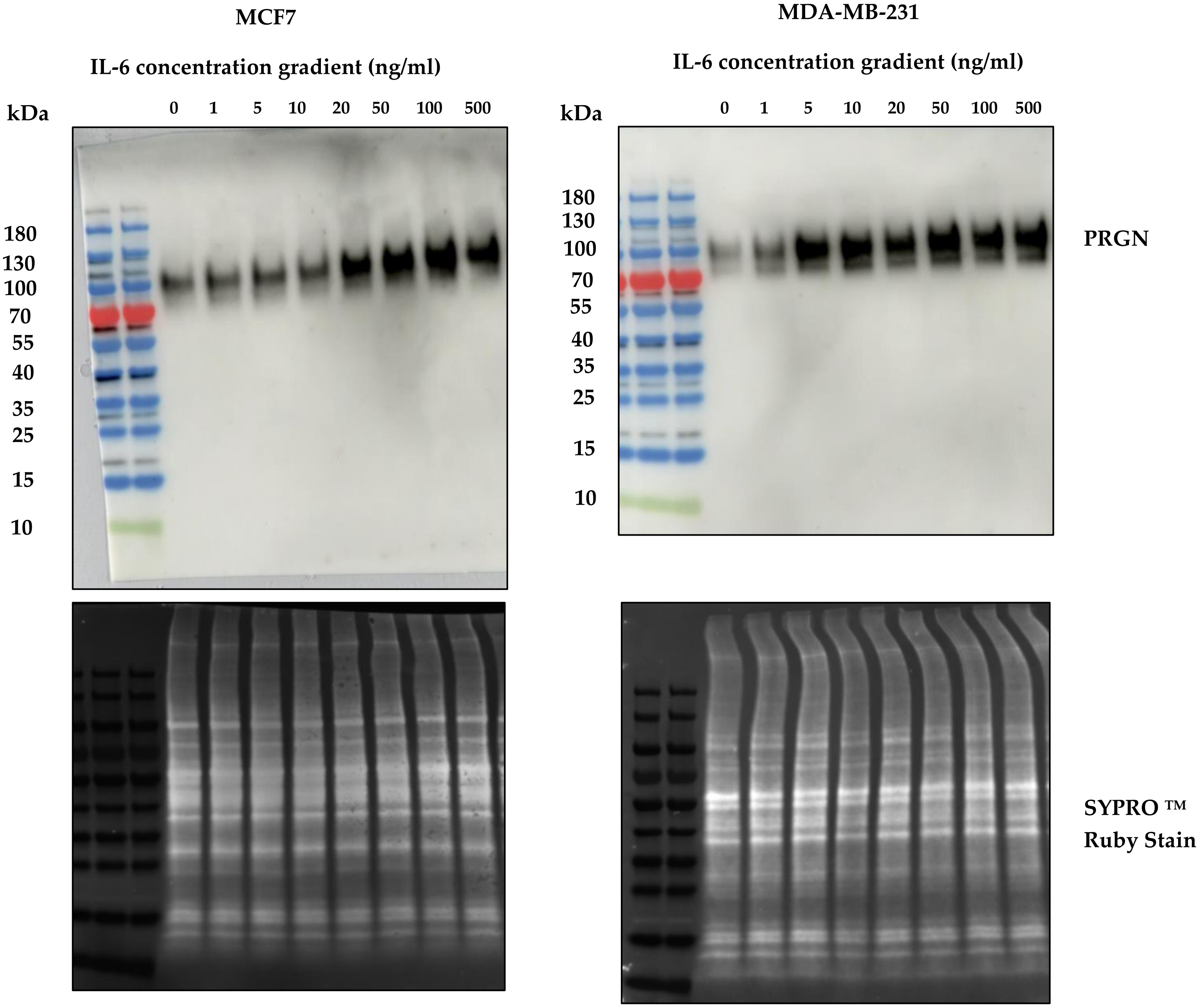
MCF7
IL-6 concentration gradient (ng/ml)
kDa
0 1 5 10 20 50 100 500
180
130
100
70
55
40
35
25
15
10
MDA-MB-231
IL-6 concentration gradient (ng/ml)
kDa
0 1 5 10 20 50 100 500
180
130
100
70
55
40
35
25
15
10
PRGN
SYPRO ™
Ruby Stain

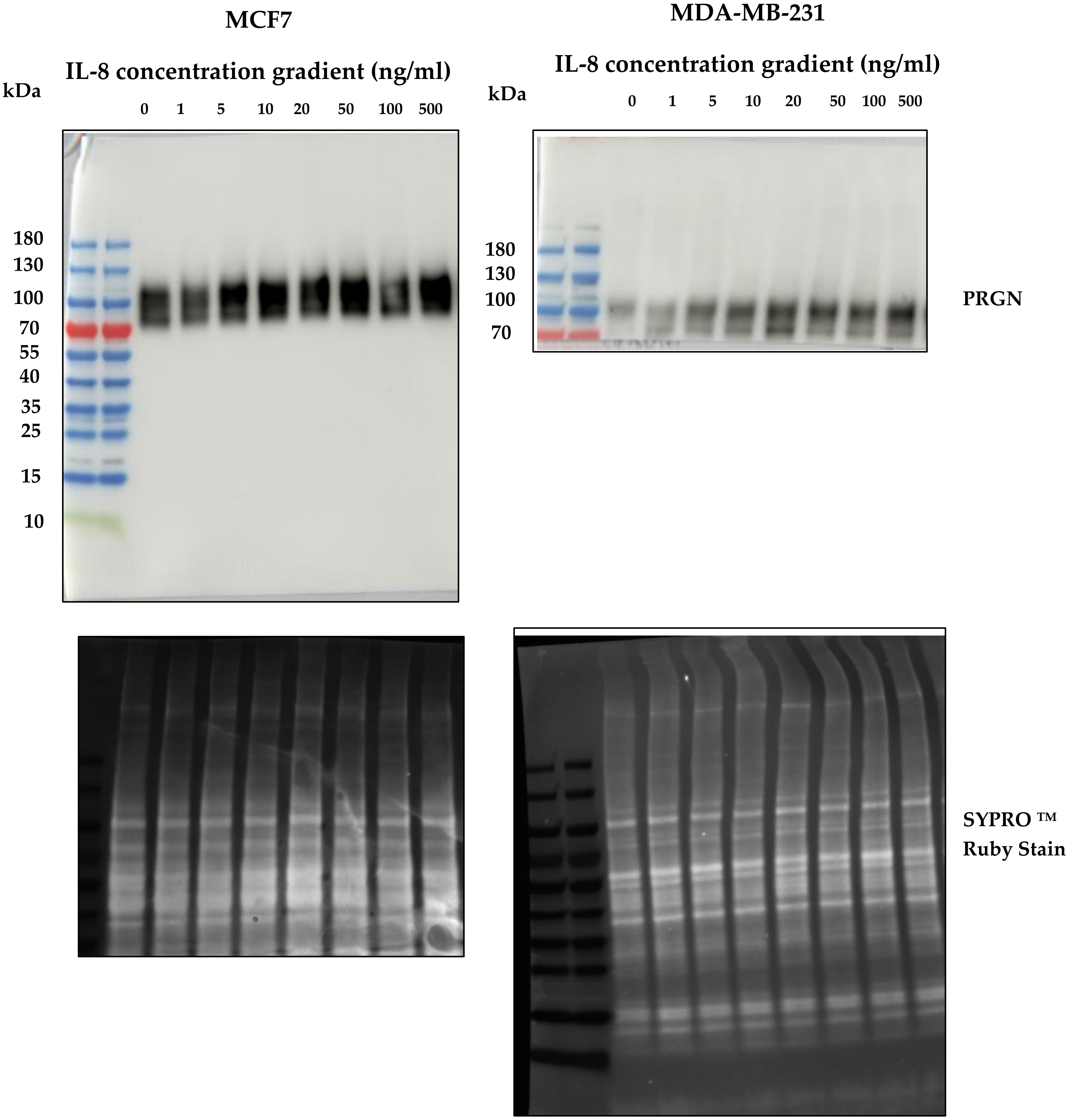
MCF7
IL-8 concentration gradient (ng/ml)
kDa
0 1 5 10 20 50 100 500
180
130
100
70
55
40
35
25
15
10
MDA-MB-231
IL-8 concentration gradient (ng/ml)
kDa
0 1 5 10 20 50 100 500
180
130
100
70
PRGN
SYPRO ™
Ruby Stain
